# Performance and acceptability of the STREAM Disinfectant Generator for infection prevention and control practices in primary health care facilities in Uganda

**DOI:** 10.1186/s13756-024-01433-1

**Published:** 2024-07-16

**Authors:** Adam Drolet, Thomas Mugumya, Shan Hsu, Jonathan Izudi, Martin Ruhweza, Emmanuel Mugisha, Rony Bahatungire, Patricia S. Coffey

**Affiliations:** 1 PATH, 2201 Westlake Ave, Seattle, WA 98121 USA; 2 PATH, PO Box 7404, Kampala, Uganda; 3 Department of Community Health, Faculty of Medicine, Mbarara University of Science and Technology, PO Box 1410, Mbarara, Uganda; 4 Uzima Ministries, PATH, Mutundwe Kirinyabigo Kampala, PO Box 7404, Kampala, Uganda; 5 Uganda Ministry of Health, Plot 6, Lourdel Road, Nakasero, Kampala, Uganda

**Keywords:** Chlorine, Environmental hygiene, Health care facilities, Infection prevention and control, Uganda, WASH

## Abstract

**Background:**

Effective infection prevention and control programs can positively influence quality of care, increase patient safety, and protect health care providers. Chlorine, a widely used and effective chemical disinfectant, is recommended for infection prevention and control in health care settings. However, lack of consistent chlorine availability limits its use. Electrolytic chlorine generators can address limited chlorine supply and stockouts by enabling onsite production of readily usable, high-quality chlorine cost-effectively. We report the feasibility (i.e., performance, acceptability, chlorine availability, and cost) of the electrolytic STREAM Disinfectant Generator (Aqua Research, New Mexico, USA) device for infection prevention and control in primary health care facilities in Uganda.

**Methods:**

We installed STREAM devices in 10 primary health care facilities in central and western Uganda. Commercial chlorine inventory records (stock cards) were reviewed in each facility to calculate average liters of chlorine received and used per month. These values were compared with actual STREAM chlorine production volumes over the study period to determine its impact on chlorine availability. We collected acceptability data from a purposive sample of device users (*n* = 16), hospital administrators (*n* = 10), and district health officers (*n* = 6) who had been directly involved in the operation or supervision of the STREAM device. We descriptively analyzed the acceptability data by user group and evaluated qualitative responses manually using a thematic approach. Cost data were normalized and modeled to determine a break-even and cost-savings analysis across a five-year period (the minimum expected lifespan of the STREAM device).

**Results:**

Chlorine was consistently available without any reported stockouts during the evaluation period. STREAM chlorine production resulted in a 36.9 percent cost-savings over a five-year period compared to commercial chlorine. User acceptability of the STREAM device was high among STREAM operators, hospital administrators, and district health officers, with all respondents reporting that STREAM moderately or significantly improved infection prevention and control practices in the health facility. Overall, 88 percent of device users and 100 percent of hospital administrators wished to continue using the STREAM device instead of commercial chlorine products.

**Conclusion:**

The STREAM device has demonstrated significant potential to strengthen infection prevention and control practices in health care facilities in Uganda. Based on the preliminary results, the STREAM device should be considered a promising tool for district hospitals and large health centers facing infection prevention and control challenges in Uganda and elsewhere, provided water and electricity are available. Going forward, implementation of the STREAM device could also be considered in smaller health care facilities in Uganda and elsewhere.

**Supplementary Information:**

The online version contains supplementary material available at 10.1186/s13756-024-01433-1.

## Background

Effective infection prevention and control (IPC) programs can positively impact the quality of health care by increasing patient safety and protecting health care providers from infection in health care facilities [1]. Health care–acquired infections (HAIs) negatively affect hundreds of millions of individuals worldwide [2, 3]. Patients in low-resource countries are at an increased risk of contracting HAIs—between 3 and 20 times higher than patients in similar settings in high-income countries [4]. Bacteria alone account for approximately 90 percent of all nosocomial infections, and protozoa, fungi, viruses, and mycobacteria account for the remaining 10 percent [5]. Most HAIs are preventable [6]. One study showed that improved cleaning practices could reduce up to 27 percent of the leading causal agents for sepsis among hospitalized patients; other pooled systematic review findings show IPC interventions could reduce HAI rates by 35 to 70 percent [6–8].

The Uganda Ministry of Health (MOH) has made strong and clear policy efforts to reduce the burden of HAIs as evidenced by the development and release of the national IPC guidelines in 2013 [9]. These policies provide a strong guiding framework, but gaps remain in their consistent and correct implementation in real-world settings. Assessments analyzing IPC capacities across the eight World Health Organization IPC Core Components highlight significant gaps in IPC education and training levels among Ugandan health staff and in supervision, monitoring, and auditing of IPC practices, along with inadequate HAI surveillance systems [10, 11]. The existing gaps in IPC programming contribute to adverse health outcomes, such as sepsis, which accounts for 10 percent of maternal deaths and 18 percent of neonatal deaths in Uganda [12, 13]. Available data show a 28 percent HAI rate in Uganda, which is substantially high [14].

Chlorine is a widely used and effective chemical disinfectant recommended for IPC in health care settings. However, despite its proven effectiveness, the lack of consistent chlorine availability and quality limits the ability of health care workers to provide a safe and hygienic environment for patients. A survey across 129,557 health care facilities in 78 low- and middle-income countries (LMICs) revealed that 36.4 percent of health care facilities lacked chlorine solution for disinfection [15]. A 2014 census of hospitals and level IV primary care facilities in Uganda showed worrying statistics regarding the availability of disinfectant solution (i.e., chlorine) for IPC: 31 percent of pediatric wards, 22 percent of post-delivery wards, and 10 percent of delivery rooms reported not having disinfectant solution [16]. Another cross-sectional study of infection control and practices in 32 health care facilities in the Arua district of the Ugandan West Nile sub-region showed that more than nine in ten (93.8 percent) of health care facilities lacked infection control committees as well as adequate supplies and equipment for infection control [17]. The notable reasons for the inadequacies in IPC practices included weak supply chains, burdensome procurement processes, and insufficient budgets [11, 18]. Combined, these factors contribute to an unsteady supply of chlorine in health care facilities, a challenge further exacerbated during times of crisis.

Electrolytic chlorine generators offer a solution to address the primary root causes of limited chlorine supply and stockouts in health care facilities, as they ensure onsite production of readily usable, high-quality chlorine in a cost-effective manner. Chlorine generators are being used to support IPC practice in more than 500 health facilities spread across 15 LMICs (Aqua Research, Inc., email communication, November 27, 2023) [19]. Previous studies conducted in Burkina Faso, Chad, and Mali illustrate how the use of onsite chlorine generators can improve the availability and quality of chlorine, as well as offer cost savings [20, 21]. Although a variety of product options exist, most are not appropriate for use in LMICs because they require large capital investment, proprietary parts, uninterrupted supply of electricity, and intensive monitoring requirements from trained technical staff operating the devices. In Uganda, chlorine generators have been piloted in health care facilities for routine disinfection needs (AquaChlor Electrolytic Chlorine Generator [Florida, USA] by UNICEF in 2021–2022 and MSR SafiStation [Washington, USA] by PATH in 2016–2019). However, uptake and use of these devices has not occurred due to the lack of commercial availability of the devices (SafiStation production was terminated in 2019) and functionality issues with AquaChlor devices (PATH/UNICEF. Report on joint monitoring visit to assess use of chlorine generators in 13 health care facilities in Uganda). To our knowledge, no evidence on the use of chlorine generators in health care facilities in Uganda has been published to date.

To overcome the challenges in IPC practices across primary health care facilities in Uganda, we assessed the feasibility of using the STREAM Disinfectant Generator (Aqua Research, New Mexico, USA), an electrolytic chlorine generator designed specifically for low-resource settings that produces a consistent flow of 0.5 percent milligram per liter chlorine solution, for IPC in ten health care facilities in Uganda.

## Methods

We used a multi-method study design to assess the feasibility (i.e., performance, acceptability, chlorine availability, and cost) of using the STREAM Disinfectant Generator device for IPC in primary health care facilities in Uganda. We collected quantitative data on STREAM device performance, STREAM and commercial chlorine availability, and cost. Qualitative feedback was also collected from device users, hospital administration staff, and key government stakeholders on STREAM acceptability. Between November 30 and December 10, 2020, STREAM devices were installed in ten health care facilities in the central and western regions of Uganda and monitored through the end of December 2021. Site selection included one regional referral hospital, three district hospitals, and six health centers. We chose to include a wide range of health levels to better understand the degree to which the STREAM device was able to address chlorine demand within different levels of the health system. According to the selection criteria we identified, the health facilities included were required to have:

- Classification as a regional referral or district-level hospital or health center IV (a county-level health facility) or health center III (a sub-county-level health facility) by the MOH.
- A current monthly commercial chlorine volume supply received and distributed within the health facility for disinfection of between 50 and 2,000 L of 0.5 percent milligram per liter solution.
- Access to a reliable supply of electricity (i.e., mains or generator).
- Up-to-date logbooks or records of commercial chlorine stock received and distributed within its units or wards.
- Approval from hospital management on taking up and operating the device.

Criteria for placement of the STREAM device within a facility included:

- A reliable source of electricity.
- Proximity to a water source.
- An area that can be subjected to spillage without disrupting services.
- A central location within the health care facility.

### STREAM device performance

We assessed the performance of STREAM devices based on whether the health facilities were able to produce equal volumes of 0.5 percent chlorine solution compared to baseline commercial chlorine stock levels, as well as the overall functionality of the ten STREAM devices installed. On each day of STREAM chlorine production, the primary device user(s) at each health facility manually tracked the volume of chlorine produced using STREAM chlorine monitoring forms. Data from these chlorine monitoring forms were collected either in person by the research team or via photos sent by the primary device user(s). In addition, the STREAM device used its built-in data logging capacity to automatically track and store hourly usage rates and provided a quantitative measure to determine total STREAM chlorine production. During monthly evaluations, the research team conducted health facility visits to retrieve data on the total operational hours recorded on the STREAM’s internal LED screen. MOH officials accompanied PATH on several of the monitoring trips as part of their oversight. The functionality status of the STREAM devices was monitored through phone calls and in-person monitoring trips by the research team. Specific device and component errors and replacement parts that had been installed were tracked for each STREAM device.

### STREAM device acceptability

We used purposive sampling to select study participants, which included: device users (health facility staff trained and responsible for STREAM operation and cleaning); hospital administrators (senior health care facility staff responsible for facility operations and involved in overseeing the STREAM operation); and government stakeholders (national governmental staff involved in the management of IPC services). The selection criteria included holding one of the enlisted roles or positions as well as being 18 years of age or older, fluent in English, and willing to provide informed consent.

We conducted key informant interviews with device users and hospital administrators using a standardized list of closed-ended and open-ended questions relating to device acceptability. National government stakeholders were asked about their overall perceptions of the STREAM device and requirements for its introduction and integration into the national public-sector health system. The interviews lasted approximately one hour, and participants received 20,000 Ugandan shillings (equivalent to US$5.61) in cellular airtime as compensation for their time. All interviews were conducted in private, well-ventilated areas and/or outside on the health facilities premises. Where needed, we adhered to the national COVID-19 protocols that necessitated social distancing and the use of personal protective equipment by both the research team and study participants.

### Commercial chlorine availability and costs

At baseline, we collected and compiled all available data from commercial chlorine inventory logs (stock cards) spanning from 80 days up to 3 years (from August 2017 to November 2020) preceding the evaluation, as well as photo images of those records. These data were used to determine and model annual chlorine volumes received and distributed within each health facility, as well as to quantify chlorine stockout frequency and duration. To model the annual chlorine volume needed, we calculated the average daily chlorine use from the days without stockouts and then extrapolated this average to cover the stockout days. The research team also collected commercial chlorine costs at baseline from the most recent expense receipts at each health facility along with the cost of water to calculate the cost of chlorine dilution for disinfection purposes. These costs were used to generate the cost of 0.5 percent chlorine solution per liter.

### STREAM device chlorine costs

To generate a STREAM chlorine cost per liter, the research team combined the Ex Works cost—cost of the device prior to transport from the manufacturer’s factory—of the STREAM device, shipping and customs duties, durable costs of materials needed for chlorine production (wooden spoon, measuring cups, 20-L bucket, and jerry can), and all consumable costs (salt, water, electricity, chlorine test strips, and vinegar) and amortized these costs over five years. The costs of electricity and water were collected from expense receipts from each health facility. In the costing analysis, we considered one Ugandan shilling as equivalent to 0.0002805 USD (US$1 = 3,565 UGX) based on the exchange rate from December 15, 2021.

### Materials

The STREAM device is a chlorine generator that uses simple inputs—salt, water, and electricity—to produce chlorine on demand, onsite. The consistent 0.5 percent milligram per liter concentration eliminates the need for chlorine dilution processes for health staff engaged in environmental cleaning. Furthermore, the simple user interface reduces the technical knowledge required to operate the device. The STREAM’s core technology received a CE marking— which stands for *Conformite Europeenne* signifying the device passed safety, health, and environmental protection requirements for sale in the European Economic Area—(No. ES151124043E) in 2016 and the power supply that converts 110/220 VAC to 12 V DC is CE-certified and IP65-rated. The 0.5 percent milligram per liter concentration of STREAM’s chlorine solution has been verified by several independent, certified laboratories (Ethiopia Conformity Assessment Enterprise, April 02, 2022, ES 877:2022; Uganda National Drug Authority, NDA/DLS/CERT/M-001–22/23, July 21, 2022; Essen & Co, March 22, 2019). Device technical specifications are noted in Table 1.

**Table 1:**
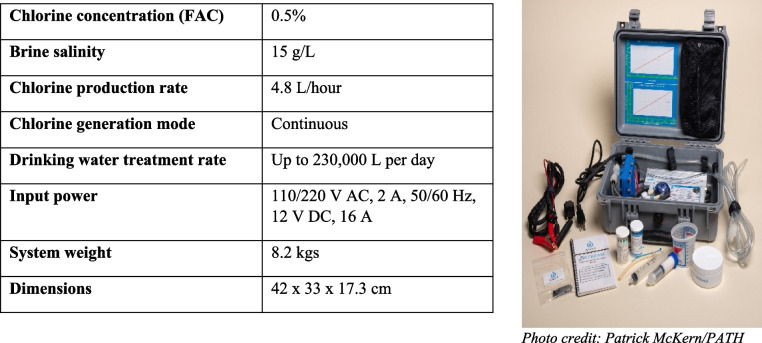
Technical specifications of STREAM device

*Abbreviation: FAC* free available chlorine

### User training

User training was led by the PATH research team in collaboration with the MOH. Primary STREAM operators—nurses, pharmacy assistants, and biomedical engineers—were identified by hospital administrators, and the training lasted about an hour. To avoid issues around STREAM operator availability for continued chlorine production, the research team trained at least three device users from each health facility on device assembly, use, maintenance, troubleshooting, cleaning, and chlorine bottling (storage). Training materials and operational guides were left at each site to serve as reference materials. Ongoing training and technical support was provided by PATH study staff through phone calls and monthly in-person monitoring visits to each health facility.

### Data analysis

STREAM chlorine production data and commercial chlorine stock inventory data were cleaned and entered into a Microsoft Excel database to generate overall and health facility–specific commercial chlorine stockout frequencies and duration, as well as a comparative analysis of chlorine volume availability across each health facility. The chlorine stock inventory data-cleaning process involved identifying handwritten records, ensuring the size and number of units of commercial chlorine were recorded correctly, and entering the data into Microsoft Excel in a consistent format, which included date, size of commercial chlorine unit, chlorine concentration, quantity of chlorine received, quantity of chlorine delivered to wards within the health facility, and total volume of 0.5 percent chlorine used. Data on STREAM device functionality and component errors were tallied in Microsoft Excel based on monthly monitoring reports.

The exploratory study was not intended to test a statistical hypothesis about the STREAM device performance but to provide descriptive findings about its functionality. Sample size for this study was purposive and not calculated to enable statistical comparisons since it was a pilot study. For the acceptability outcome of this evaluation, we calculated frequencies and proportions, stratified by user group for quantitative data. Analysis of qualitative responses to open-ended questions was conducted manually using a thematic analysis approach. Data matrices were used to examine differences by level of health care and respondent type. Microsoft Excel (Washington, USA) was used to manage both quantitative and qualitative data. We present qualitative findings using themes.

Commercial chlorine costs and STREAM-related costs were normalized and analyzed to determine comparative per**-**liter cost savings estimates for each health facility and by health facility level and to generate five-year break-even analyses, which is the minimum expected lifespan of the STREAM device.

### Ethical review and approval

Ethical review and approval for the acceptability portion of the evaluation that included human subjects was obtained from the Mulago Hospital Research Ethics Committee (MHREC Reference #2076, April 22, 2021) and the Uganda National Council of Science and Technology (UNCST Reference #HS1467ES, July 28, 2021). We obtained administrative clearance from the MOH on September 25, 2020 (Reference #ADM/214/283/01). We obtained unwitnessed verbal consent from all participants, which was documented in the project database. A consent form describing the assessment procedures, risks, and possible benefits was read in English. All participants were informed that their participation was voluntary and that they could decline to answer any question. All stakeholder interviews were conducted in a private setting to ensure confidentiality.

## Results

Ten STREAM devices were installed in ten health care facilities in the western and central regions of Uganda between November 30 and December 12, 2020, following national COVID-19 preventative measures and protocols. Table 2 shows the distribution of the STREAM devices and study sites.

**Table 2:** Health care facilities where STREAM device was used, by type and location

Facility level	Western region	Central region	Total
Regional referral hospital	0	1	1
District hospital	1	2	3
Health center IV	2	1	3
Health center III	3	0	3
**Total**	6	4	10

Within the health facilities, the STREAM devices were installed in the maternity ward (*n* = 4), pharmacy (*n* = 2), outpatient department (*n* = 1), immunization room (*n* = 1), surgical theater (*n* = 1), and equipment maintenance workshop (*n* = 1). A total of 24 primary users were trained across all ten health facilities.

### STREAM performance

**Fig. 1:**
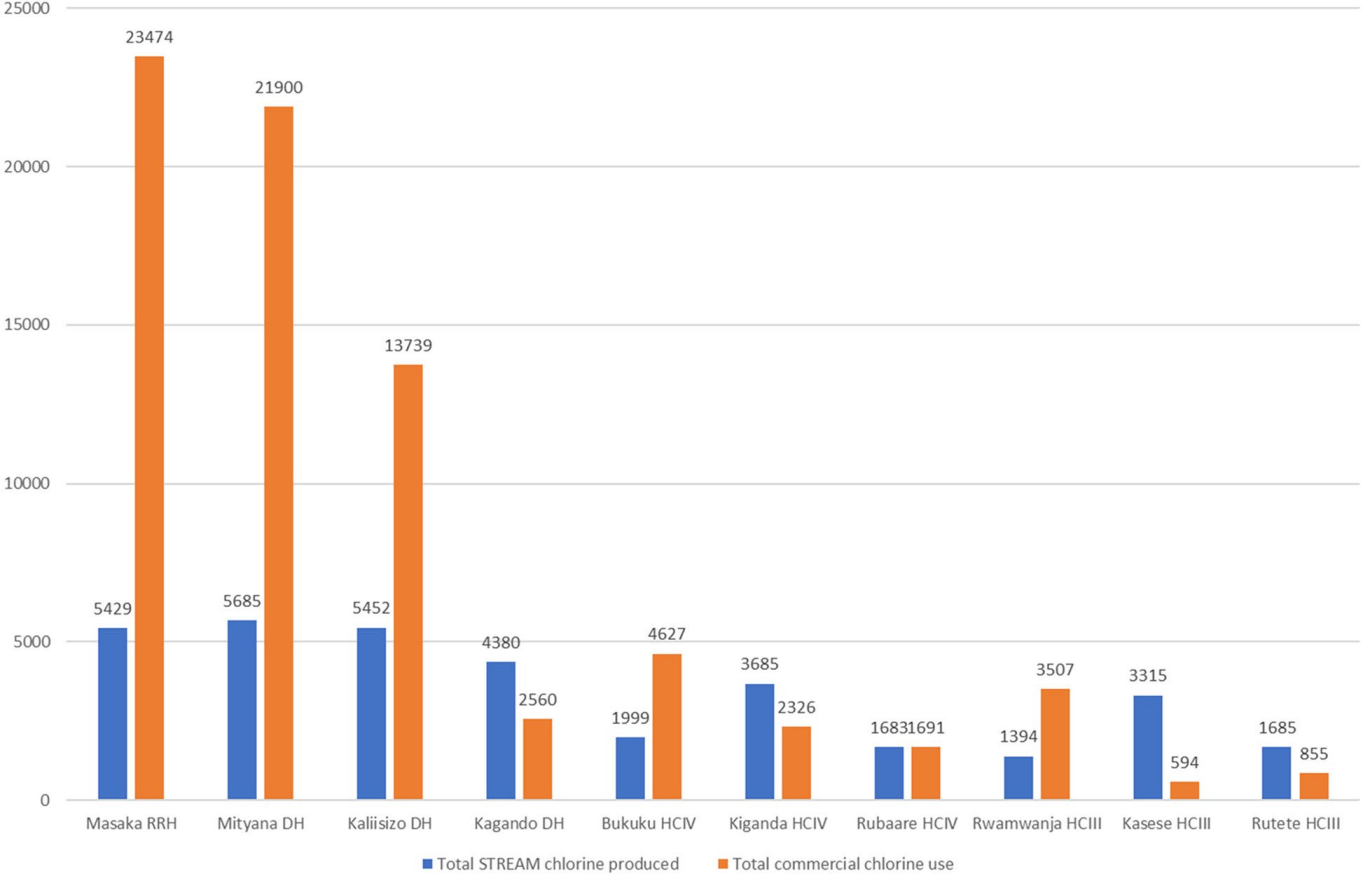
Cumulative volume (liters) of 0.5 percent STREAM chlorine produced compared to annual commercial chlorine stock volumes. *Abbreviations:* *RRH* regional referral hospital, *DH* district hospital, *HCIII* health care III facility, *HCIV* health care IV facility, *RRH* regional referral hospital

Overall, 34,975 L of 0.5 percent chlorine disinfectant were produced by the STREAM units from November 30, 2020, to December 12, 2021, translating to a total value of 22,360,201 UGX (US$6,272; December 2021). Across the ten primary health care facilities, five had higher annual chlorine volumes produced with the STREAM devices compared to the annual baseline commercial chlorine stock levels (Fig. 1). No chlorine stockout was reported at any of the ten health facilities.

Only two STREAM devices experienced no technical issues or component errors during the study period. The remaining eight STREAM devices experienced one or more errors, such as leaking reaction chambers, faulty power supplies, and issues with circuit boards (Table 3). These errors were due to contextual factors such as power surges in mains (electricity sources) and high calcium content in source water, as well as manufacturing issues like a weak seal on the reaction chamber and weak socket connections in circuit boards. A majority (65 percent) of the STREAM component errors occurred in three of the ten health facilities. These results led Aqua Research to redesign the power supply, circuit board, and reaction chambers in late 2021. Replacement components were shipped in November 2021 and installed in all devices in December 2021.

**Table 3:** Summary of STREAM component errors, causes, and mitigation approaches

Issue type and description	Cause	Solution
**Manufacturing and contextual:** Leaking reaction chamber	Scaling in the reaction chamber or outlet port due to calcium in the water clogging and building up pressure inside the cell, which led to leaking.	• An emergency pressure-relief rupture disk was added to the brine inlet port of the cell. The rupture disk provides a point of pressure relief for the cell when the cell needs to be cleaned. • An outer titanium plate was added to the cathode housing to prevent the cathode housing from warping (where the oxidant was leaking). • Cleaning protocols were updated.
**Manufacturing:** Poorly soldered circuit board connections	Energy demands require more robust, higher-amp socket connectors.	• All socket connectors were replaced with stronger, more robust, click-to-connect connectors that prevent accidental shorts and keep wires from becoming dislodged. • The connector between the power supply and control box was upgraded to a 42A connector to prevent overheating.
**Contextual:** Power supply vulnerability to voltage spikes	Severe power surges led to tripped thermal switches and damaged power componentry.	• Aqua Research redesigned the power supply to include a 120-277 V surge protector and added a 42A connector from the power supply to the control box.

### STREAM device acceptability

We collected acceptability data from a purposive sample of device users (*n* = 16), hospital administrators (*n* = 10), and district health officers (*n* = 6). The overall user acceptability of the STREAM device was high as shown in Table 4. Three key themes around usability and satisfaction emerged from the feedback: 1) ease of STREAM operation and impact on health care worker workload; 2) ease of STREAM integration and contribution to infection prevention and control practices in the health facility; and 3) willingness to continue using the STREAM over commercial chlorine.

**Table 4:** Acceptability of STREAM device for infection prevention and control in health care facilities, by device users (*n* = 16)

	**Very easy**	**Moderately easy**	**Neutral**	**Moderately challenging**	**Very challenging**	**Comments**
Ease of device operation	63%	19%	13%	6%	0%	“Almost everything is ok. I just dissolve salt in water, switch on the machine and go away and leave it producing chlorine.” DU02 HCF09
Ease of cleaning device	50%	25%	19%	6%	0%	“It [cleaning] only required vinegar no other complicated procedures.” DU01 HCF06
Ease of integrating the STREAM into current IPC practices	81%	13%	6%	0%	0%	“Staff get enough jik [chlorine] as they want and they don’t make requisitions to stores which involves paperwork.” DU01 HCF02
	**Very confident**	**Moderately confident**	**Neutral**	**Moderately concerned**	**Very concerned**	
Confidence that other health workers will be able to correctly operate the STREAM	75%	19%	6%	0%	0%	“Because operating procedures are clear, short and precise.” DU01 HCF01 “Colored pictures are welcome. The instructions should be pasted on the wall for easy reference.” DU01 HCF10
	**Significantly increased**	**Moderately increased**	**No change**	**Moderately decreased**	**Significantly decreased**	
Change in workload compared to previous chlorine preparation and use practices	38%	19%	6%	13%	25%	*Increase* “Increased demand from hospital departments.” DU01 HCF01 “Disinfection is done on a daily basis compared to before when it was done after 3 days.” DU01 HCF03 *Decrease* “It gives chlorine that does not need dilution therefore it reduces time taken diluting.” DU01 HCF09 “No need to dilute for surfaces compared to the commercial chlorine.” DU02 HCF03 “Less requisitions for jik [chlorine] from store.” DU01 HCF06
	**Significantly improved**	**Moderately improved**	**No change**	**Moderately worsened**	**Significantly worsened**	
Effect of STREAM use on IPC practices in the health facility	81%	19%	0%	0%	0%	“There has been constant supply of chlorine at the facility.” DU02 HCF03 “It has augmented supply of chlorine during times of scarcity so that we don’t use plain water and soap only.” DU01 HCF08 “Increased availability of chlorine thus improved cleanliness hence patient safety.” DU01 HCF08 “Every corner is cleaned with enough chlorine, less risks for acquiring infections.” DU01 HCF06

*Abbreviations*: *DU* Device user, *HCF* Health care facility, *IPC* Infection prevention and control

### Ease of STREAM operation and impact on health care worker workload

STREAM operators (82 percent) and hospital administrators (100 percent) found the STREAM very or moderately easy to use. Fifty percent of device users reported a reduction in IPC-related workload, as a result of not having to dilute the STREAM’s 0.5 mg per liter concentration for disinfection purposes. Conversely, 44 percent of STREAM users noted an increase in their IPC workload, which was attributed to the time required to operate the device and troubleshoot errors. Device users also noted that they disliked having to use the STREAM chlorine within 24 h of production to avoid issues with chlorine concentration degradation, as well as disliking the frequent cleaning of the device. Interestingly, each device user who noted the STREAM increased workloads also mentioned the increase in chlorine availability led to improved IPC practices.

*“Disinfection of surfaces is done on a daily basis compared to before when it was done after 3 days.”DU01 HCF03*

Hospital administrators reported several benefits and challenges associated with STREAM operation by health staff. The ability to generate chlorine onsite and on demand was a consistent theme noted by hospital administrators, along with the simple processes required to operate the STREAM device. Hospital administrators also noted a few challenges with the STREAM performance, production capacity, and chlorine shelf life:

*“We need to have people always operating it in order to produce chlorine otherwise if you switch it on and go away, you may find very little jik [chlorine] produced because it can go off or get a fault.” HA01 HCF04*

### Ease of STREAM integration and contribution to IPC practices in the health facility

All device users, hospital administrators, and district health officers reported the STREAM moderately or significantly improved IPC practices in the health facility. The STREAM was seen as moderately or very easy to integrate into IPC practices by 94 percent of device users, 90 percent of hospital administrators, and 100 percent of district health officers. Across all three respondent groups, three primary benefits to IPC practices were mentioned as a result of STREAM use: improved availability of chlorine and addressing chlorine stockouts; elimination of chlorine dilution processes; and improved cleanliness, odor, and patient safety. As reported by two hospital administrators, STREAM positively impacted patient care and environments in addition to staff workload:

*“Improved availability of jik [chlorine] which makes it possible for us to implement infection control practices more effectively, especially in theatre and maternity. We have reduced post-operative wound sepsis because we have used a lot of chlorine produced to clean the theatre and ensure it is well disinfected.” HA01 HCF10*

*“Since we started using chlorine from the STREAM, the labor ward stopped smelling which was not the case with the previous supplies. It cleared away every bad smell.” HA01 HCF04*

### Willingness to continue using the STREAM over commercial chlorine

Overall, 94 percent of device users and 100 percent of hospital administrators (*n* = 10) wished to continue using the STREAM over commercial chlorine. Five of the six district health officers (83 percent) were very supportive of introducing the STREAM into the national public health system in Uganda. Improving the speed of chlorine production from the STREAM and providing additional training and training material, as well as greater maintenance and troubleshooting support, were noted by all three respondent cohorts as factors that would improve the overall usability, functionality, and experience.

*“Have people with technical know-how visiting periodically and help with maintenance so that it's on all the time.” HA01 HCF03*

### Commercial chlorine availability

At baseline, we collected and compiled all available data from commercial chlorine inventory logs (stock cards) spanning from 80 days to 3 years (from August 2017 to November 2020) preceding the evaluation, as well as photo images of those records. During the year immediately preceding the installation of the STREAM devices, across all ten health facilities, an average of 2.5 periods of stockout of chlorine supply (each lasting about 30 days) were identified. On average, health facilities had chlorine stock during 74 days (approximately 2.5 months) per year. The average duration that the health facilities operated without any chlorine stock ranged from 25.8 to 132.3 days (approximately 1 to 4.5 months). Health center IIIs and IVs reported the longest duration of chlorine stockout (Table 5).

**Table 5:** Chlorine stockout length and frequency prior to STREAM device installation

	**Average stockout length (days)**	**Average number of stockouts per year**	**Total days per year without chlorine**
Health center III (*n* = 3)	40.1	2.0	80.2
Health center IV (*n* = 3)	44.1	3.0	132.3
District hospital (*n* = 3)	10.3	2.5	25.8
Regional referral hospital (*n* = 1)	29.5	2.2	64.9
Overall average (*n* = 10)	29.7	2.5	74.3

### Cost of chlorine produced

A comparison of the costs required to generate 34,975 L of 0.5 percent chlorine with the STREAM device compared to commercial chlorine costs showed that health facilities would expect an average cost savings of 36.9 percent per liter when using the STREAM device (Table 6). Commercial chlorine costs included the actual chlorine cost paid by the facility and water costs for chlorine dilution. STREAM chlorine costs were calculated as the sum of capital cost and operational recurrent cost. Capital costs include the costs of the STREAM device, device shipping, customs duties and taxes, wooden stir spoon, measuring cup, jerry can, and bucket. STREAM operational cost includes the costs of salt (15 g per liter of 0.5 percent chlorine solution produced), water, electricity, and vinegar (0.5 L per cleaning cycle). See Supplemental Table 1 for detailed costing data.

**Table 6:** Costing of commercial versus STREAM chlorine production

Description	Commercial chlorine	STREAM	Cost savings (US$) (%)
Total cost of 34,975 L of 0.5% chlorine volume produced (December 2020 through December 2021)	$6,272 22,360,201 UGX	$3,958 14,110,165 UGX	$2,314 (36.9%) 8,250,036 UGX
Average cost per liter of 0.5% chlorine	$0.1793 639 UGX	$0.1132 403 UGX	$0.0662 (36.9%) 236 UGX

Exchange rate on December 15, 2023: US$1 = 3,565 UGX; 1UGX = US$0.0002805

## Discussion

Pragmatic solutions, such as onsite chlorine generation, offer innovative approaches for strengthening IPC in health care facilities in low- and middle-income countries [22]. Global recommendations highlight chlorine as an effective, intermediate-level disinfectant for routine and terminal cleaning of areas such as high-touch surfaces, floors, and blood (or other bodily fluid) spills [23, 24]. Expanding IPC assessment tools, such as the World Health Organization’s IPC assessment framework and Water and Sanitation for Health Facility Improvement Tool (WASHFIT) to include indicators on chlorine or disinfectant stock availability is needed to better quantify and identify chlorine supply gaps in health facilities [25, 26]. Chlorine offers several advantages to health staff including its broad spectrum (sporicidal) efficacy, rapid inactivation effect, low cost, and wide availability [27, 28]. With an increasing number of studies showing evident gaps in chlorine availability and access across primary health care settings (combined with the emergence of a greater array of disease threats), decentralized, easy-to-use, cost-effective IPC solutions should be emphasized [29, 30].

The findings from this evaluation of a novel electrolytic chlorine generator in Ugandan health care facilities demonstrate promise for the integration and rollout of the STREAM device across health care facilities in Uganda and settings with similar IPC challenges. The STREAM device enabled health staff to overcome supply chain issues that often led to chlorine stockouts by producing quality chlorine, on demand and onsite. Our study results—particularly the feasibility and willingness for onsite chlorine production in primary health care facilities—reflect similar findings from a project led by Catholic Relief Services in Burkina Faso, Ghana, and Liberia where 7,305 L of 0.5 percent STREAM chlorine were produced across 14 STREAM devices and distributed to 103 health facilities in the three countries [31]. Similarly, a study in Chad involving 67 chlorine generators in health facilities found that users generated 100 percent of their chlorine demand five months after device installation [32]. With the continued use of these devices, a greater focus will be needed on long-term maintenance and repair to ensure sustained use and chlorine production.

Acceptability, particularly among frontline health staff, and value for money are two factors that are critical for the sustained adoption of health interventions. Our study demonstrated consensus among STREAM users, hospital administrators, and district health officials, as all reported that the STREAM increased chlorine availability, leading to a perception of strengthened IPC practices in the study health facilities. Future implementation will need to carefully consider how to manage and minimize increases in health staff workload resulting from STREAM use and avoid contributing to staff burnout [33, 34]. Overall, health staff reported that the use of STREAM chlorine led to perceived safer and cleaner patient environments, simplified chlorine procurement and distribution processes, and cost savings.

Health administrators across all levels of the health system rely on financial and economic cost information to make evidence-based health policy decisions [35]. Our study also produced key costing evidence for health system leaders, illustrating how the STREAM could be a valuable, cost-saving investment for health care facilities in Uganda. These results are similar to a STREAM cost-savings analysis across eight health facilities in Ghana that reported a 47.5 percent cost reduction when using the STREAM [36]. Combined, these results demonstrate how onsite chlorine production addresses supply chain and chlorine availability issues faced by primary health facilities.

Supported by the results of this study, in June 2022, the National Drug Authority approved a certificate of analysis (NDA/DLS/CERT/M-001–22/23) verifying the chlorine produced from the STREAM device complies with British Pharmacopia and United States Pharmacopia specifications for the respective tests done, and in October 2022, the National Advisory Committee on Medical Equipment (NACME) recommended the STREAM device for health centers and district hospitals in Uganda (RSPS 21 10 2221 10 22). Currently, the MOH is developing a national STREAM scale-up strategy and district-level implementation plans to introduce and scale up STREAM devices throughout Uganda’s health care system.

### Strengths and limitations

Our evaluation has several strengths and some limitations. To the best of our knowledge, this is the first published evaluation to focus on the feasibility of using a STREAM device for onsite chlorine generation at primary health care facilities in Uganda. Our acceptability data drew from participants directly engaged in IPC practices along with their managers, lending credibility to our findings. We provide data that can set a benchmark for future implementation science research and full economic evaluation studies focused on STREAM devices in Uganda. The potential limitations of this evaluation include the small sample size and narrow geographic scope of the intervention. A longer evaluation period and detailed focus on device reliability would generate greater insights into the long-term functionality of the device. However, this is a question for future research. The overlap between the study period and the 2020 SARS-CoV-2 pandemic may have influenced results on chlorine availability, use, and stockouts, given the increased demand for chlorine for infection prevention and control practices. Further analysis of chlorine demand and availability beyond the COVID-19 pandemic period and across a larger sample of health facilities would further contextualize our study results. Our costing analysis provides valuable insights into potential cost savings within different health facility levels and at different chlorine demand levels; however, the analysis did not include the full range of costs associated with total cost of ownership, such as installation, operation, maintenance, and repair of the devices. Further exploration using implementation research to assess health system fit—including assessing how the STREAM can be used as an entry point for conducting IPC assessments, launching IPC training for health staff, and improving documentation of IPC supplies and practices—may be helpful, along with a more thorough cost-effectiveness or total cost of ownership analysis [27]. Consistent with the results of a recent systematic review of interventions to improve water supply and quality, as well as sanitation and handwashing facilities in health care facilities, and their effects on HAIs in low- and middle-income countries, additional research around the impact of consistent availability of cleaning products, and chlorine in particular (both as a standalone intervention and as part of an IPC bundle) on reducing health care–associated infections in low- and middle-income countries should be explored and could reduce any potential courtesy bias in acceptability feedback [37].

## Conclusions

During the evaluation period, chlorine was consistently available and no chlorine inventory stockouts were reported. The use of the STREAM device for chlorine production led to a 36.9 percent cost savings compared to commercial chlorine products in primary health care facilities. Future costing analysis should account for the total cost of ownership of the STREAM device by including the aforementioned factors. User acceptability of the STREAM device was high, and all hospital management staff at both the health facility and district levels perceived the STREAM as positively contributing to improved IPC practices at their health facilities. Overall, almost all device users and all hospital administrators wished to continue using the STREAM as opposed to commercial chlorine. These preliminary results demonstrate that the STREAM device has potential to strengthen infection prevention and control practices in district facilities and health centers that have access to water and electricity in Uganda. Going forward, implementation of the STREAM device could also be considered in smaller health care facilities facing similar infection prevention and control challenges in Uganda and elsewhere, provided water and electricity are available. The present evidence should be considered preliminary data for future studies.

### Supplementary Information

Supplementary Material 1.

## Data Availability

The datasets used and/or analyzed during the current study are available from the corresponding author on reasonable request.

## Abbreviations

DU: Device user
FAC: Free available chlorine
HA: Hospital administrator
HCIII: Health center III
HCIV: Health center IV
HCF: Health care facility
IPC: Infection prevention and control
RRH: Regional referral hospital
MOH: Uganda Ministry of Health
WASHFIT: Water and Sanitation for Health Facility Improvement Tool
