# Supplementary material for: Performance and acceptability of the STREAM Disinfectant Generator for infection prevention and control practices in primary health care facilities in Uganda
Source: Antimicrob Resist Infect Control. 2024 Jul 16;13:77. doi: 10.1186/s13756-024-01433-1 (PMC11251206; doi:10.1186/s13756-024-01433-1)
Supplement: Supplementary file 1 — Supplementary Material 1. [file 13756_2024_1433_MOESM1_ESM.docx]

Supplemental Table 1: STREAM capital and recurrent costs used in cost calculations

|  | USD | UGX |
| --- | --- | --- |
| Commercial chlorine 3.5% per 5L |  | 38,800 |
| STREAM Capital cost | | |
| STREAM Device | $2,650 |  |
| Shipping, duties and taxes | $160 |  |
| Wooden stir spoon |  | 20,000 |
| Measuring cup |  | 7,000 |
| Bucket (20L) |  | 25,000 |
| Jerry can (20L) |  | 8,800 |
| STREAM Recurrent cost | | |
| Vinegar per L |  | 18,000 |
| Water cost per m3 |  | 0 – 1,050 |
| Salt cost per 0.5kg (15g per L of chorine produced) |  | 1,900 |
| Electricity cost per kWh |  | 0 – 1,880 |
